# Construction of nanocarriers based on nucleic acids and their applications in nanobiology delivery systems

**DOI:** 10.1093/nsr/nwac006

**Published:** 2022-01-17

**Authors:** Yingshu Guo, Xiuping Cao, Xiaofei Zheng, Sk Jahir Abbas, Juan Li, Weihong Tan

**Affiliations:** Shandong Provincial Key Laboratory of Molecular Engineering, School of Chemistry and Chemical Engineering, Qilu University of Technology (Shandong Academy of Sciences), Jinan 250353, China; School of Chemistry and Chemical Engineering, Linyi University, Linyi276005, China; School of Chemistry and Chemical Engineering, Linyi University, Linyi276005, China; Institute of Molecular Medicine, State Key Laboratory of Oncogenes and Related Genes, Renji Hospital, Shanghai Jiao Tong University School of Medicine, College of Chemistry and Chemical Engineering, Shanghai Jiao Tong University, Shanghai200240, China; Institute of Cancer and Basic Medicine, Chinese Academy of Sciences, The Cancer Hospital of the University of Chinese Academy of Sciences, Hangzhou310022, China; Institute of Cancer and Basic Medicine, Chinese Academy of Sciences, The Cancer Hospital of the University of Chinese Academy of Sciences, Hangzhou310022, China

**Keywords:** nanocarriers based on nucleic acids, biosensing, targeted delivery, cell

## Abstract

In recent years, nanocarriers based on nucleic acids have emerged as powerful and novel nanocarriers that are able to meet the demand for cancer-cell-specific targeting. Functional dynamics analysis revealed good biocompatibility, low toxicity and programmable structures, and their advantages include controllable size and modifiability. The development of novel hybrids has focused on the distinct roles of biosensing, drug and gene delivery, vaccine transport, photosensitization, counteracting drug resistance and functioning as carriers and logic gates. This review is divided into three parts: (i) DNA nanocarriers, (ii) RNA nanocarriers and (iii) DNA/RNA hybrid nanocarriers and their applications in nanobiology delivery systems. We also provide perspectives on possible future directions for growth in this field.

## Teaser

This review summarizes the recent advances of nanocarriers based on nucleic acids and their biological applications, providing perspectives on possible future directions for growth in this field.

## INTRODUCTION

Cancer treatment still relies heavily on chemotherapy, and despite substantial progress we still see severe damage to normal cells and various side effects, including pain, for patients [1–4]. To overcome these obstacles, scientists are eagerly pursuing drug delivery strategies that are safer and more selective. In particular, the use of nanocarriers based on nucleic acids (NCNAs), as nanopharmaceutical carriers have attracted increasing attention owing to their ability to overcome many existing limitations. Progress has been made in elucidating their performance [5–8]. NCNAs are a class of nanostructures containing nucleic acids (DNA, RNA or both). They include nucleic acid self-assembly origami and nucleic-acid-decorated nanoparticles (NPs). They strictly follow the principle of complementary base pairing, allowing easy construction of a targeting nanocarrier by changing the order or type of base [9–12]. Nucleic acids can be produced easily and quickly by commercial DNA and RNA synthesizers. Therefore, NCNAs have become a highly promising nanomaterial for use in the field of nanomedicine.

To date, researchers have designed many different nanocarriers, such as gold NPs combined with a very wide range of inorganic systems [11,13–15], biofilm-modified nanoplatforms [16] and liposome-based systems obtained through organic synthesis [17,18]. The nanocarriers mentioned above have achieved much in the field of nanomedicine, but they also face some limitations in medical development. For example, cationic polymer surfaces are cytotoxic, dendritic polymer nanocarriers are prone to increased immunotoxicity *in vivo* and inorganic NP residues in the body are difficult to catabolize [3]. Therefore, the low toxicity and high programmability of nucleic acids have attracted increasing attention, and a series of self-assembled NCNAs have been screened for bioimaging, cargo loading and transport, diagnostic logic gates, and other fields [4,19–28]. In addition, as biological macromolecules, nucleic-acid-based nanomaterials can be combined with various nanomaterials to form multilevel nanopharmaceutical carriers [29,30]. The NCNAs base can be assembled through a biomimetic drug-loading system, improved with erythrocyte membranes [16], modified by peptides or active proteins [26,31,32], incorporated into nanomaterials for aptamer assembly and used in drug-loading systems based on siRNA [10].

NCNAs can facilitate surface functionalization. The nucleic acids, a type of biomacromolecule, are self-assembled from the ATCGU nucleotides, strictly following the Watson-Crick pairing principle. The number and position of each atom and bond are clearly defined, giving nucleic acids atomic-level precision and the ability to precisely self-assemble. The spatial addressability of NCNAs can locate the position of molecules by adjusting the sequence. Accordingly, aptamers and nucleic acid nanostructures allow functional molecules such as aptamers, folic acid and bovine serum albumin to be precisely integrated into specific locations of NCNAs [1]. For example, NCNAs with modified transferrin can specifically recognize the transferrin receptor on cells, resulting in strong targeting [32]. Hence, researchers can build NCNAs with multiple functions, such as precise identification, targeted delivery and intelligent control of drug release [24,25].

**Figure 1.**
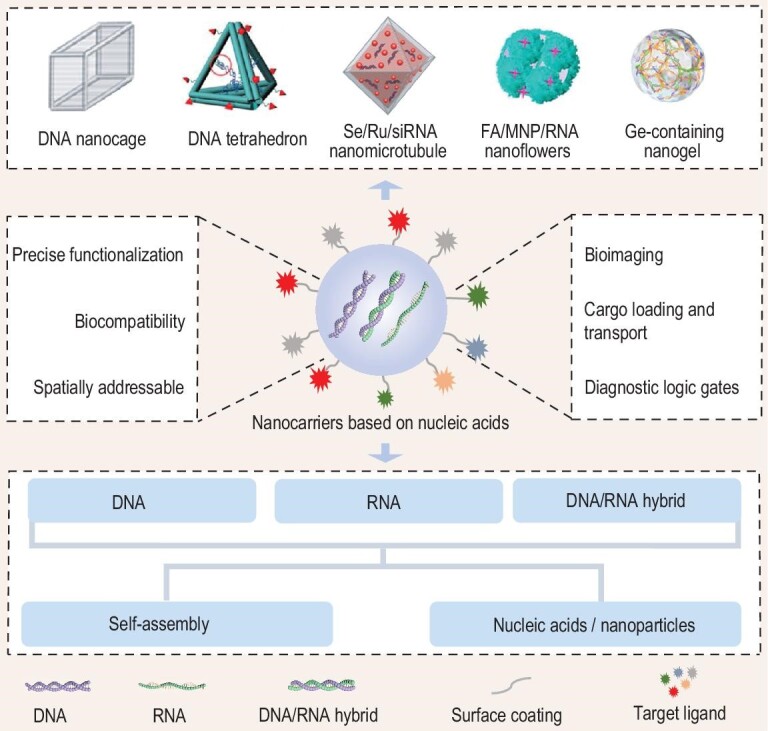
NCNAs for application in nanobiology delivery systems. NCNAs are divided into DNA nanocarriers, RNA nanocarriers and DNA/RNA hybrid nanocarriers. These nanostructures are constructed by methods such as self-assembly and combination with nanoparticles, including DNA nanocage [78], DNA tetrahedron [49], Se/Ru/siRNA nanomicrotubule [67], FA/MNP/RNA nanoflower [69] and Ge-containing nanogel [48]. DNA nanocage reprinted with permission from ref. [78]. Copyright 2020, Springer Nature. DNA tetrahedron reprinted with permission from ref. [49], Copyright 2018, American Chemical Society. Se/Ru/siRNA nanomicrotubule reprinted with permission from ref. [67]. Copyright 2017, American Chemical Society. FA/MNP/RNA nanoflower reprinted with permission from ref. [69]. Copyright 2017, American Chemical Society. Ge-containing nanogel reprinted with permission from ref. [48]. Copyright 2019, American Chemical Society.

In this review, we consider the latest developments in NCNA systems. We attempt to illuminate the properties of DNA and RNA nanocarriers and the merits of NCNAs. We summarize the latest progress on NCNAs in the field of nanomedicine, focusing on biocompatibility, targeting capability, programmability etc. and looking at applications in biosensing, cargo transport and logic gates (Fig. 1).

## DNA NANOCARRIERS

As the basis of life, DNA molecules offer diverse biological characteristics, such as programmability, low toxicity, biocompatibility and selectivity [33–35]. Double-stranded DNA hybridizes in strict accordance with the principle of base pairing, forming a double-helix-like antiparallel structure. Therefore, desired nucleic acid structures can be designed by changing the type or sequence of bases. Increasing interest in DNA nanotechnology has opened doors in the field of nanomedicine [36].

Since DNA materials are synthesized according to the principle of base complementation, we can use this principle to design various DNA sequences. The use of a DNA synthesizer can make the process simple and easy, safe and controllable. DNA has the characteristics of programmability, sequence specificity and spatial addressability and can accurately functionalize NCNAs to realize the recognition and detection of individual molecules [37–40]. DNA nanocarriers are herein classified into two categories—self-assembled DNA and NPs decorated with DNA—and they play important roles in various chemical reactions, bioimaging and targeted delivery [41–44].

**Figure 2.**
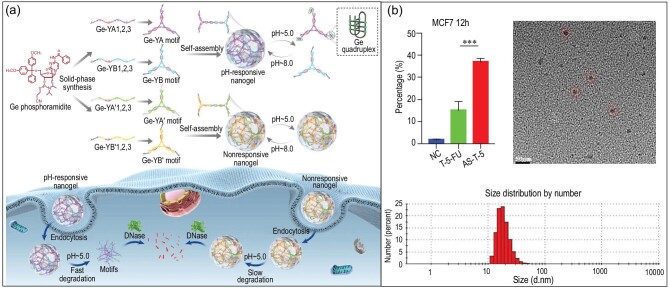
Construction and experimental characterization of DNA nanocarriers. (a) Schematic illustration of Ge-containing DNA nanogel self-assembly and acid-triggered disassembly of the pH-responsive nanogel and intracellular drug release process. Reprinted with permission from ref. [48]. Copyright 2019, American Chemical Society. (b) The cytotoxicity and characterization of a DNA tetrahedron loaded with 5-fluorouracil. Reprinted with permission from ref. [21]. Copyright 2019, American Chemical Society.

DNA can be used to construct multicomponent nanocarriers by hybridization, covalent bonding or coupling to different materials, such as NPs [13–15,45], proteins [22], peptides [46,47] and liposomes [17,18]. During synthesis, gemcitabine was used in the deoxycytidine state based on its similar functionality [48]. The DNA sequence containing Ge is used as the synthetic raw material for the construction and functionalization of the vector through hybridization. It is well known that this type of cytosine-rich DNA nanostructure can respond to pH. Therefore, NCNAs can realize drug delivery and intelligent drug release through changes in environmental pH (Fig. 2a). This method of constructing DNA nanostructures by hybridization is relatively simple and has been widely used. In recent years, researchers have used similar principles to design spherical nucleic acids (SNAs), complex DNA tetrahedrons [21,49,50], DNA prisms [12], DNA nanocages [51] and some self-assembled complex nanomaterials [23,52–54]. The typical DNA tetrahedron has high assembly efficiency and structural rigidity, which also means high stability and structural controllability, and has non-negligible potential in gene regulation, drug delivery, biological imaging, targeted recognition and other aspects. A classic example is the DNA tetrahedron constructed by Zhan *et al*. for drug delivery during cancer therapy [21]. The DNA tetrahedrons were co-incubated with the cells for 12 hours, and drug intake in the cells was analyzed by flow cytometry. The data showed that the percentage of drugs in cells was nearly 40%, which fully proved the stable and efficient drug delivery ability of the DNA tetrahedron (Fig. 2b). SNA is a kind of nanomaterial that has attracted increasing attention in recent years. Rolling circle amplification (RCA) is a common method for constructing DNA nanocarriers, and SNA with a predetermined sequence was synthesized by researchers using the RCA technique [55]. Based on the precise functionalization of DNA, SNA was combined with magnetic NPs (MNPs) to form NCNA, which was applied in the field of biological imaging. However, there are still some problems with this SNA construction method. Typically, SNAs are constructed using monolayers of dsDNA or ssDNA as the carrier, a design that limits the therapeutic efficacy of the drugs and prevents the scaling up of their production. Therefore, Li *et al*. proposed a new strategy for the construction of DNA nanocarriers, namely, the self-assembly of two alternating short DNA blocks and the preparation of long-strand DNA nanostructures by supersandwich hybridization. The SNA carrier loaded with a large number of double-stranded structures provides the same number of doxorubicin (DOX) loading sites, which greatly improves the drug-carrying capacity of the nanocarrier [11]. The combination of these nucleic acid nanostructures with gold NPs further increases the rigidity of nanomaterials and reduces the toxicity and side effects of chemical drugs on normal tissues and cells [11,37]. DNA nanocarriers have produced good results in the stable delivery of drugs and the improvement of drug delivery efficiency, but there are still aspects to be further improved. For example, at present, many nanocarriers still exhibit insufficient selectivity and limited application effects. The existence of DNA adapters can solve this problem to a certain extent. Although the screening process of aptamers is complex, they are easily internalized *in vivo*, and their modification can effectively improve the targeting of nanocarriers and reduce off-target side effects [56,57]. Some researchers conducted experiments using aptamers. For example, the cyclic bivalent aptamer designed by Zhou *et al*. showed high stability and strong targeting [44]. In addition, another significant task is to modify bifunctional aptamers to NPs, which enables the nanosome to respond to both target cells and ATP molecules in target cells at the same time, thus further improving the accuracy of targeting [58]. Undoubtedly, this kind of aptamer-modified DNA nanostructure has great application prospects with regard to improving selectivity. In conclusion, functionalized DNA nanocarriers have the ability to accurately deliver drugs to targets, tightly encapsulate drugs and effectively reduce toxicity and side effects of drugs *in vivo* and *in vitro*. However, in terms of structural control and performance enhancement of DNA nanomaterials, we believe that further development is needed [43,59].

## RNA NANOCARRIERS

Similar to DNA, RNA is an important biological macromolecule in nature that guides protein synthesis [60]. It also plays a role in carrying the genetic information of certain bacteria and viruses and is a promising nanomaterial [26,61]. RNA is a single strand transcribed with one strand of DNA as a template. By changing the sequence and type of bases, researchers can design and adjust the overall structure of RNA nanocarriers [7,8]. Unlike DNA, RNA nanotechnology can transcend the limitations of the double helix structure to form a large variety of structures and multiple types of circular-structured modules, facilitating the design of a series of complex and controllable nanocarriers [62]. In addition to the three main types of RNA, namely, rRNA, tRNA and mRNA, many other RNAs comprise the RNA family. In recent years, the role of RNA interference mechanisms caused by small-molecule RNA in tumor treatment has attracted the attention of many researchers [63]. When foreign genes, such as viral genes and transposons, enter cells, they usually produce double-stranded RNA. Endonucleases in cells immediately cut them into siRNAs with a specific structure and size. Antisense siRNA combines with certain enzymes to form a complex that not only cleaves homologous single-stranded RNA but also binds to the target RNA to produce more double-stranded RNA under the action of the enzyme until the mRNA is completely eliminated [64]. With this principle, we can use RNA-based vectors to inject specific dsRNA into cells for tumor therapy. This is more effective, more accurate and faster than traditional methods of specifically suppressing target genes and can be designed to address different needs. Different dsRNA and RNA nanocarriers have met the demands of personalized treatment [65]. There are two main ways to build RNA nanocarriers: RNA self-assembly and RNA decoration on NPs, which are both now widely used in biological imaging, drug delivery and gene silencing [66].

**Figure 3.**
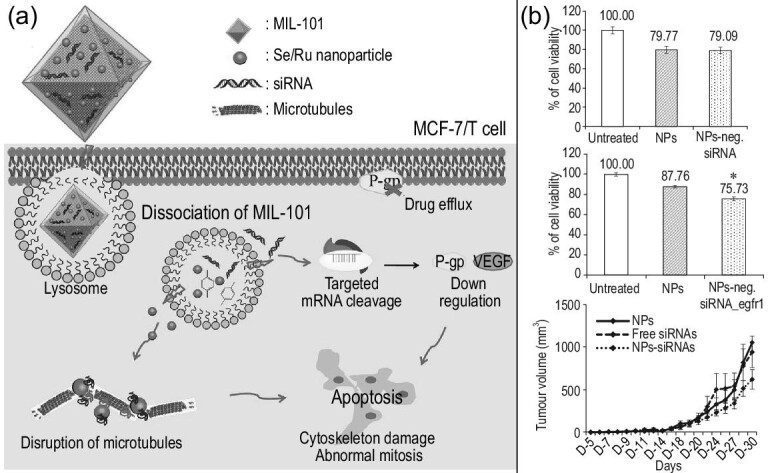
Examples and validation of RNA nanocarriers. (a) RNA nanocarrier structure and mechanism of action for gene therapy. Reprinted with permission from ref. [67]. Copyright 2017, American Chemical Society. (b) The therapeutic effect of RNA nanocarriers *in vivo* and *in vitro*. Reprinted with permission from ref. [70]. Copyright 2017, Informa UK Limited, trading as Taylor & Francis Group.

To circumvent the defects in chemotherapy, we look to new strategies for constructing nanocarriers with high selectivity and low cytotoxicity. The excellent biocompatibility of RNA and the unique effect of RNA interference have attracted increasing attention [61]. RNA can be used as a modified material, but also as small RNAs *in vivo* to silence specific genes, especially those related to target tumors, and effectively inhibit tumor growth and spread [9,26]. RNA is unstable and easily degraded in the blood. To address this problem, researchers have combined RNA with polyethylene glycol [8], inorganic NPs [61,67], glue bundles [4], carbon nanospheres [68], peptides [31], proteins [32] and other materials to form RNA composite nanocarriers. For example, MNPs were introduced into an RNA nanofluorescence agent to form MNP/RNA-NF, followed by modification with folic acid (FA). Then, RNA-NF was combined with DOX and a photosensitizer to form nanomaterials (FA/MNP/RNA-NF/D/T) [69]. This RNA composite carrier had high selectivity and stability, and it protected its RNA components from degradation. In addition, increasing attention has been given to the advantages of combining RNA with metal NPs, inorganic NPs and other traditional NPs. Traditional NPs have led to remarkable progress in the field of nanomedicine, but they also inevitably have limitations. However, the combination with RNA breaks through these limitations, and the RNA nanocarriers formed have higher selectivity and better therapeutic effect, truly achieving the effect of 1 + 1 > 2. For example, a nanocarrier composed of Se/Ru metal-organic NPs and siRNA effectively enhanced cell uptake and promoted siRNA escape from endosomes/lysosomes under surface coordination. It plays a synergistic role in the treatment process to kill cancer cells [67] (Fig. 3a). Some researchers have tried to use pH-sensitive carbonate apatite NPs and siRNA to form nanodrug loading systems, another effective way to prevent drug resistance. A similar example is the combination of apatite NP carbonate with siRNA [70]. We observed that this RNA composite vector had stronger cytotoxicity than NPs in the cell activity assay. Both *in vivo* and *in vitro* experiments showed that the RNA vectors were more satisfactory (Fig. 3b). We believe that RNA nanomaterials will play an important role in future nanomedicine [71].

## DNA/RNA HYBRID NANOCARRIERS

Another class of NCNAs is nanocarriers based on DNA/RNA hybrids. These nanocarriers contain both DNA and RNA, which is a relatively common method of constructing and functionalizing nanocarriers. Researchers designed a folate-derived DNA dendrimer nanocarrier combined with anti-HuR siRNA [72]. This carrier had the fastest synthetic approach and high selectivity in targeted tumor therapy *in vivo*, making further study worthwhile. In addition, Ruan *et al*. introduced SNA nanocarriers with siRNA grafted onto their spherical surface [73]. These nanocarriers were quickly absorbed by more than 60 kinds of cells and protected siRNA from enzymatic digestion, enabling safe delivery to cells. The siRNA broke out under the cleavage of an intracellular Dicer enzyme and exerted an effect on cancer cells. This type of DNA/RNA nanocomposite can also be used to load chemotherapeutic drugs while exerting gene therapy effects. Under the premise of fulfilling the expected function, the toxicity can be minimized.

**Figure 4.**
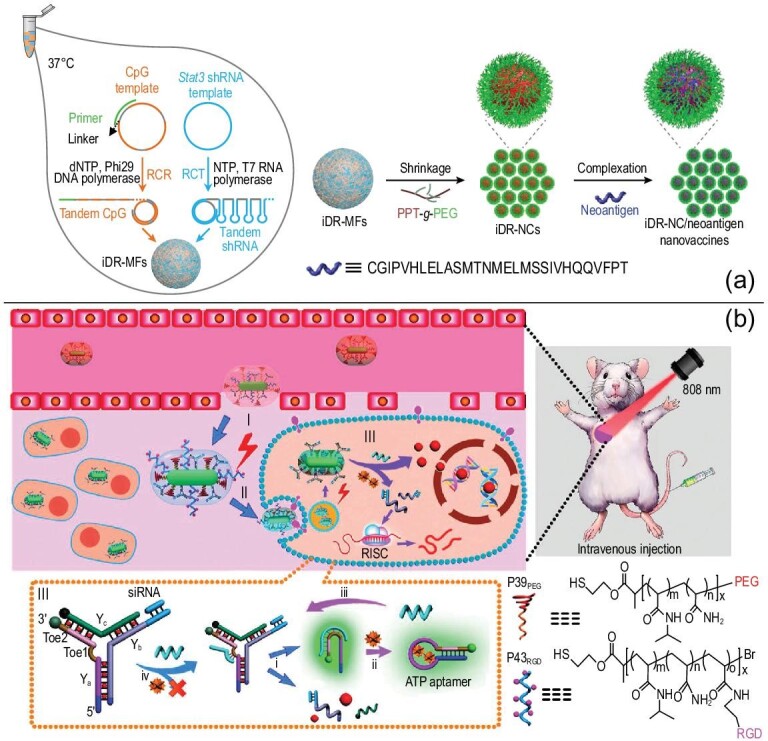
Schematic diagram of the construction of DNA/RNA hybrid nanocarriers. (a) Schematics of DNA/RNA hybrid nanovaccines for synergistic tumor immunotherapy. Reprinted with permission from ref. [74]. Copyright 2017, Springer Nature. (b) Schematic design of the smart nanocarriers triggered by miRNA and fueled by ATP. Reprinted with permission from ref. [75]. Copyright 2016, American Chemical Society.

The precise design of NCNAs can fully reflect the advantages of nucleic acids in functional regulation. The precise functionalization of NCNAs by DNA/RNA hybridization can prevent the residual toxicity and side effects of some nanocarriers, and realize the construction and application of more intelligent drug carriers. For instance, Zhu designed nanocapsules formed by the self-assembly of DNA/RNA in the same system [74]. In addition to ensuring biosafety, this biologically stable NCNA can effectively co-deliver and induce T cell memory, which has great potential in tumor therapy (Fig. 4a). In addition, the combination of DNA/RNA and NPs provides the potential to construct intelligent nanomaterials. Intelligent DNA/RNA nanocarriers with responsive and active targeting are helpful for drug accumulation and release at target sites—for example, NCNAs composed of DNA nanorobots, gold NPs and RNA accumulated in the tumor site, and controlled drug release *in vivo* under the guidance of near-infrared (NIR) light [75] (Fig. 4b). This intelligent nanocarrier is obviously in line with the needs of modern nanomedicine. We believe that DNA/RNA nanocarriers will show increasingly broad prospects.

## BIOLOGICAL APPLICATIONS OF FUNCTIONALIZED NCNA$\bf{s}$

NCNAs provide various prospective advantages, including low toxicity, biocompatibility and high selectivity. They have led to intensive research in nanomaterial technology, resulting in progress in bioimaging, cargo loading and transport, and the diagnostic application of logic gates [76].

### Bioimaging

**Figure 5.**
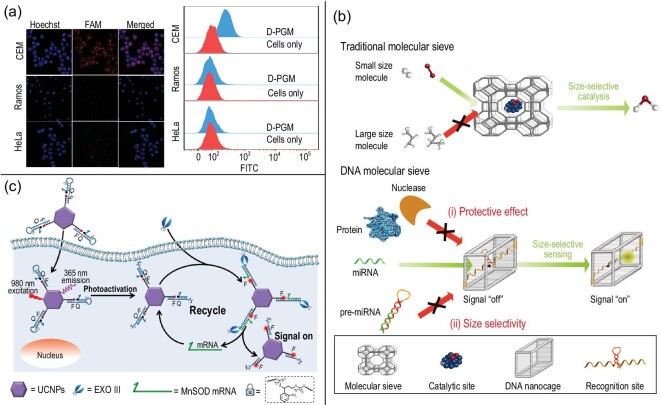
Construction methods and functions of different types of NCNAs in biosensing. (a) D-PGM constructed by self-assembly method for cell imaging. Reprinted with permission from ref. [27]. Copyright 2020, American Chemical Society. (b) Construction of DNA molecular sieve. Reprinted with permission from ref. [78]. Copyright 2020, Springer Nature. (c) Schematic diagram of nucleic acid nanomaterials combined with upconversion NPs for signal-amplified mRNA imaging. Reprinted with permission from ref. [79]. Copyright 2020, American Chemical Society.

Bioimaging is a common application of NCNAs. Researchers have used DNA nanofluorophores, which can effectively cross the blood-brain barrier and have been used to enhance the fluorescence imaging of brain tumors in the NIR-II region, and innovatively combined the DNA amphiphilic block polymer PS-b-DNA as a nanocarrier for organic luminescent NPs [77]. Compared with the commonly used PS-b-PEG carrier, PS-b-DNA has better performance in tumor imaging, diagnosis and treatment, but needs further study. NCNAs have also been explored for biological detection and tracking. For example, the nanocarrier DNA precision guided missile (D-PGM) was loaded with fluorescent substances and DOX. The fluorescence property of D-PGM allows the trajectory of the drug to be tracked [27]. Through confocal fluorescence imaging experiments, the CEM, Ramos and HeLa cells treated with D-PGM were observed to show high-intensity fluorescence. A flow cytometric assay was used to detect CEM, Ramos and HeLa cells treated with D-PGM, which showed obvious fluorescence (Fig. 5a). Obviously, D-PGM has excellent capabilities in biological imaging. In Fu's research, nucleic acid nanotechnology was applied to increase accuracy. Nucleic acid probes were encapsulated in the internal cavity of the backbone nucleic acid. Small target molecules could enter the cavity for effective molecular recognition, while large molecules could not [78] (Fig. 5b). This system not only delivered mature microRNA and precursor microRNA into cells, but also effectively prevented nuclease degradation. In biosensors, with the expansion of spatiotemporally controllable signals, the amplification method can control the time and amplification process, enabling sensitive mRNA imaging of live cells selected at specified intervals of the cell life cycle. Duan's research work used NIR light to precisely control and trigger the light-controlled nucleic acid amplification of the entire process [79] (Fig. 5c). DNA-based nanotechnology enables us to identify and analyze target materials more accurately and effectively in the field of biosensors. It is clear that NCNAs have potential that is worthy of further exploration and application [55].

### Cargo loading and transport

#### Chemotherapy drug

**Figure 6.**
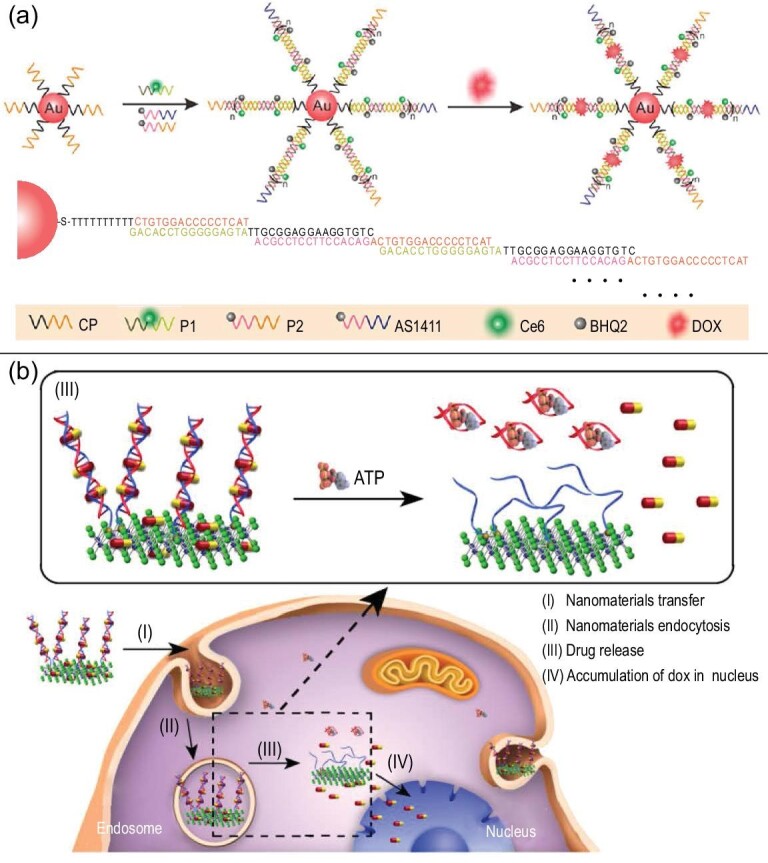
The structure and drug delivery process of different types of NCNAs. (a) Schematic illustration of the synthetic process of Ce6-PSNA-Dox-AS1411. Reprinted with permission from ref. [11]. Copyright 2018, American Chemical Society. (b) Schematic illustration showing the intracellular processes of drug delivery and ATP-induced release from DOX/D1/MoS_2_-NS. Reprinted with permission from ref. [37]. Copyright 2017, American Chemical Society.

Traditional chemotherapy and radiation therapy are forms of cancer treatment with the disadvantage of inevitably causing severe damage to normal tissue cells. Functionalized NCNAs have very high selectivity, which can address this issue. In recent years, functionalized NCNAs have been widely used in combination with peptides, chemotherapy drugs and other substances to achieve controlled release with changes in magnetic, photothermal and pH conditions [80]. A new SNA vector was modified by aptamer AS1411 to enter tumor cells by nucleoside-mediated endocytosis, after which the structure changed, releasing the loaded drug through interaction with ATP [11]. Furthermore, this spherical DNA polymer combined with gold NPs through short-distance self-assembly. With a supersandwich hybridization reaction to increase drug loading, it was considered a promising drug delivery vehicle (Fig. 6a). However, there is still much room for optimization of SNA construction technology. DNA-functionalized MoS_2_ nanomaterials [64] provided another vector construction idea. This DNA/MoS_2_/aptamer delivered the drug DOX to the target cell and disintegrated under stimulation by ATP (Fig. 6b). This NCNA achieves accurate release in drug delivery and serves as a typical demonstration for the construction of stimuli-responsive NCNAs. In addition, there are many amazing NCNA designs for drug delivery, such as DNA nanocages containing DOX [51] and DOX ampules [23].

#### Genes

Gene silencing is a phenomenon in which genes cannot be expressed or are insufficiently expressed in organisms for some reason, and it is also a mechanism for removing abnormal RNA from eukaryotic cells. The common principle of siRNA-mediated gene silencing is the same as that of the RNA interference effect described above. siRNA combines with proteins and enzymes to form a silencing complex. The helicase divides RNA into a sense strand and an antisense strand. The sense siRNA binds to the target mRNA to degrade it and synthesize new siRNA, while the direct mRNA is completely eliminated after circulation. The rational use of gene silencing can target the intended gene more accurately and achieve faster and more effective treatment of cancer. Gene silencing technology has a wide range of applications in nucleic acid nanomaterials.

**Figure 7.**
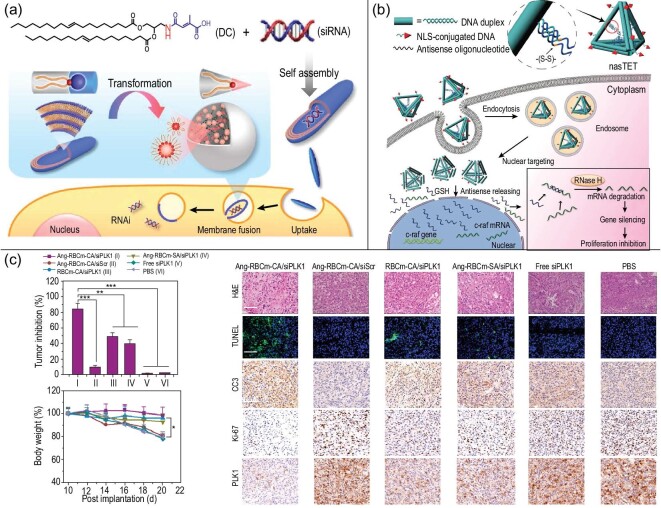
The functionalization of NCNAs and their application in gene delivery. (a) Schematic illustration of the synthesis of nanotransformers and the delivery mechanism. Reprinted with permission from ref. [81]. Copyright 2018, American Chemical Society. (b) Structure of a multifunctional double-bundle DNA tetrahedron and its cellular uptake fate. Reprinted with permission from ref. [49]. Copyright 2018, American Chemical Society. (c) *In vivo* experiments of gene delivery through NCNAs to explore therapeutic effects and biological safety. Reprinted with permission from ref. [16]. Copyright 2020, American Chemical Society.

However, because of the short half-life of siRNA in the blood, researchers have designed NCNAs to ensure the integrity of siRNA and the smooth progress of gene silencing [81,82]. He *et al*. proposed a lipid-bound NCNA system that is degradable in acidic environments, which not only greatly promotes endosomal siRNA escape but also prevents unnecessary drug release in normal tissues *in vivo* [81] (Fig. 7a). The structure is simple and easy to control. One drawback is that lipid-based nanomaterials tend to be large, unstable and easily dispersed drug molecules. Accordingly, NCNA with a DNA tetrahedron as the main body seems to be a better choice. DNA tetrahedrons containing nucleo-targeting peptides and antisense oligonucleotides enter the cell in a more stable state to silence proto-oncogenes, increasing nuclear and cytoplasmic downregulation by selecting target mRNAs [49] (Fig. 7b). In contrast, the NCNA structure and size are more controllable.

We know that the strategy of using chemicals as drugs for tumor treatment is widely applied. In comparison, gene therapy is more targeted and has biological safety advantages with which chemotherapy cannot compete. Researchers used NCNAs to deliver genes into mice for treatment. In comparison with the control group, they showed that this delivery strategy can effectively inhibit tumor growth. The weights of the mice in the experimental group did not fluctuate much, which proved that the toxicity and side effects were not obvious. The use of H&E, TUNEL apoptosis, cleaved caspase 3, proliferation and PLK1 staining further proved the excellent biosafety of NCNA [16] (Fig. 7c). Obviously, the application of NCNAs in the field of gene delivery has achieved good results, and nucleic acids are a nanomaterial category worthy of further attention [83].

#### Vaccines

Using the body's immune system to treat cancer is a strategy worth trying. We used NCNAs to deliver vaccines into organisms and kill cancer cells through the immune system, thereby achieving tumor treatment. Liposome spherical nucleic acids (LSNAs) have many advantages, such as adjustable chemical structure and rapid access to cells without the aid of transfectants. They are also an attractive platform for gene regulation and immunomodulation therapy. Researchers have designed two functional LSNA vectors [5]. The new form of LSNA exhibited faster cell uptake and higher sequence-specific flux after entering the cell, achieving functions such as receptor activation, which made it a promising candidate for immunotherapy and for the use of vaccines in new treatment approaches. However, we look forward to building a more efficient and convenient NCNA. Researchers used lipid-modified nucleotides binding to DNA strands to form micelle nanocarriers of uniform size [17]. In a separate self-assembly step, micelles could be equipped with immunoadjuvant agents (CpGs), and fluorescent probes of DNA NPs could be made into multifunctional carrier systems for *in vivo* and *in vitro* immune stimulation. The dose-dependent activation of splenic dendritic cells (DCs) by CpG and NP was accompanied by a significant upregulation of costimulatory molecules and cytokines. Therefore, researchers were inspired to use the carrier to transport a vaccine into the body to stimulate an immune response against a tumor. In Yoshizaki's research, two compound methods (premixing and postmixing) were used to match Toll receptor 9 in DC endosomes [18]. The combination of CpG-DNA and mGlu-HPG modified cationic liposomes constituted a drug delivery system that further activated antigen-specific immunity. These liposomes could promote the production of DC cytokines and the expression of costimulatory molecules *in vitro* to induce antigen-specific immune responses *in vivo*. Compared with traditional pH-sensitive polymer-modified liposomes, premixed and postmixed liposomes had a stronger antitumor effect. The study also confirmed the importance of designing effective vaccine vectors by using appropriate CpG-DNA coordination methods.

**Figure 8.**
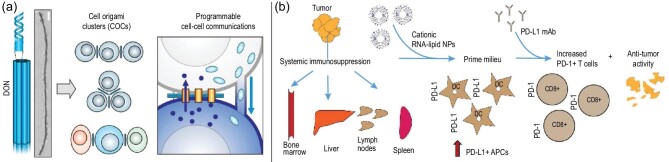
NCNAs are used for vaccine delivery. (a) DNA origami nanostructure (DON)-based biomimetic membrane channels to organize cell origami clusters (COCs) with controlled geometric configuration and cell–cell communications. Reprinted with permission from ref. [84]. Copyright 2021, American Chemical Society. (b) Scheme of RNA-NPs triggering an immune response. Reprinted with permission from ref. [34]. Copyright 2018, American Chemical Society.

As mentioned above, NCNAs exhibit extraordinary performance in the field of vaccine delivery. Researchers have continued to explore and have produced a new idea. Based on the DNA origami nanostructure, a bionic membrane channel was designed to organize cell origami clusters (COCs) with controlled geometry and intercellular communication [84] (Fig. 8a). T cells and cancer cells were assembled in a certain ratio and structure, and the resulting COCs could carry out immune responses *in vitro*. This research provides a novel strategy for cancer immunotherapy. Another classic example is mRNA lipid-NP vaccines, which can activate systemic and intratumoral myeloid cells [34]. RNA-NPs entering the tumor induce PD-L1 to trigger a wide range of immune responses (Fig. 8b). From this perspective, the use of NCNA-based immunotherapy as an effective and safe treatment is worthy of further study.

#### Photosensitizers

NCNAs can be used for photodynamic therapy (PDT) or photothermal therapy (PTT). NCNA-based phototheranostics have superior performance in terms of safety, adaptability and selectivity.

**Figure 9.**
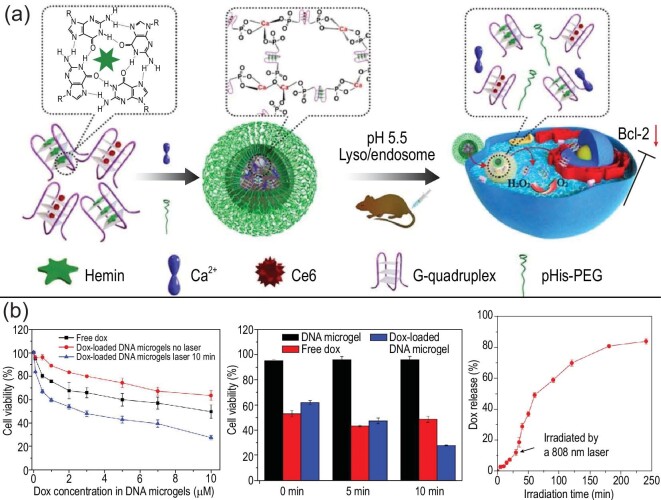
Representative examples of NCNAs for photosensitizer delivery. (a) A schematic illustration for synthesis of Ca-AS1411/Ce6/hemin@pHis-PEG. Reprinted with permission from ref. [85]. Copyright 2018, American Chemical Society. (b) Drug release efficiency of NIR-light-responsive magnetic DNA microgels and their effect on cell viability. Reprinted with permission from ref. [43]. Copyright 2017, American Chemical Society.

PDT uses a laser of a specific wavelength to illuminate a photosensitizer. Photosensitizers transfer energy to surrounding oxygen, producing highly reactive singlet oxygen, which is cytotoxic to tumor cells and kills them. At present, some researchers have explored the application of NCNAs in PDT therapy, such as the combination of NCNAs containing photosensitizers and porphyrins containing iron [85] (Fig. 9a). This kind of NCNA can overcome the limitation of hypoxic conditions and better exert the PDT effect to kill tumors. In addition, a novel NCNA consisting of smart copper (II), gold NPs and platinum has been designed, which will be combined with PDT and PTT therapy to significantly improve efficacy [86]. Better still, it can work against a wide range of tumors without concerns about drug resistance. In the field of nanomedicine, the application of NCNAs in PDT is undoubtedly a direction worth exploring.

The goal of PTT is to deliver high-efficiency photothermal conversion materials to the body. These materials are concentrated near the tumor, and when irradiated by an external light source, they convert light energy into heat energy, thereby killing cancer cells. We know that using heat to kill cancer cells requires a very high temperature. When PTT is applied directly to the human body, high temperatures can cause serious harm to patients. So the researchers engineered NCNA with an outer layer of dopamine. It not only delivers siRNA safely to the target, avoiding degradation by enzymes, but also produces PTT effects at relatively low temperatures and achieves good therapeutic effects, killing two birds with one stone [87]. This provides a strategy worth considering for killing tumors at relatively low temperatures. In addition, NCNAs with potential for PTT therapy could also be endowed with the ability to deliver drugs intelligently. For example, DNA-modified double-reaction microgels control the release of drugs under the guidance of NIR light [43]. The results show that the DNA-modified microgel can control the drug release rate under NIR light, and the longer the irradiation time, the higher the drug release rate. This conclusion was further confirmed by cell experiments (Fig. 9b). In a more complex effort, Wang *et al*. have designed a double-reactive microgel that converts light energy into heat when applied to NIR light, not only enabling the carrier to effectively release its payload, but also killing cancer cells using the light-induced effect. We believe that the application of NCNAs in phototheranostics provides a new technology for the construction of intelligent nanomaterials and the diagnosis and treatment of tumors, which is worth looking forward to.

#### Codrugs

**Figure 10.**
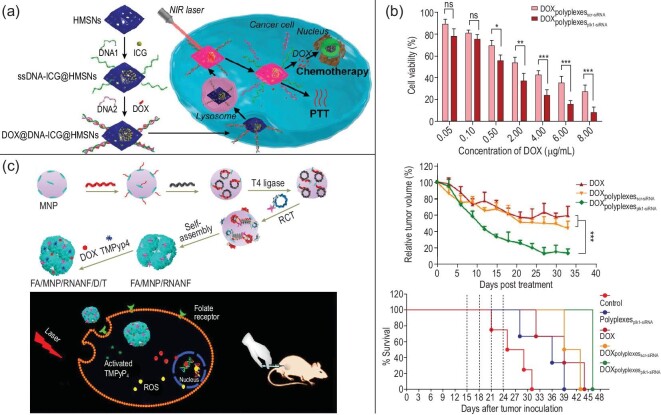
Example and validation of NCNAs for codrug delivery and enhanced therapeutic effect. (a) Schematic demonstration of the synthesis of multifunctional DOX@DNA-ICG@HMSNs and their utilization in NIR-laser-controlled chemo-photothermal combined therapy of neoplastic cells. Reprinted with permission from ref. [80]. Copyright 2018, American Chemical Society. (b) Exploration of the therapeutic effects of different groups in the combined treatment strategy. Reprinted with permission from ref. [2]. Copyright 2017, American Chemical Society. (c) FA- and biotin-modified RNA NF, self-assembled on the surface of an MNP via hybridization, and schematic diagram of the effect in the cell. Reprinted with permission from ref. [69]. Copyright 2017, American Chemical Society.

To kill cancer cells more safely and effectively, we often use a variety of mechanisms for combined treatment, so researchers have extensively explored the use of NCNAs to transport codrugs. Codrugs exert a synergistically therapeutic effect on tumors in the body through a variety of mechanisms and do not easily cause drug resistance while killing cancer cells. As shown in Fig. 10a, Hai *et al*. constructed an NCNA for codrug delivery [80] based on NIR light-sensitive DNA-modified hollow mesoporous silica NPs loaded with indocyanine green (ICG) and DOX. The NCNA releases ICG and DOX into target cells to produce chemotherapy and photothermal therapy triggered by NIR light. Compared with a single treatment strategy, this collaborative treatment method has a stronger killing effect on cancer cells. From this perspective, this superior treatment effect has also played a certain role in avoiding the problem of drug resistance caused by poor treatment effects. In addition, many researchers have explored the therapeutic effects of a combination of multiple drugs. A typical example is a composite nanosystem that simultaneously loads DOX and polo-like kinase I (plk1) siRNA [2]. In cell experiments, with increasing DOX content, the effect of this synergistic treatment became more obvious. The drugs were injected into mice. Then it was observed that the tumors in the treatment group containing both DOX and plk1-siRNA were significantly inhibited in each control group, and the survival rate of the other groups was significantly higher than that group (Fig. 10b). In addition, the FA-modified magnetic RNA nanoflowers (RNA NFs) proposed in Guo's research work were combined with the anticancer drug DOX and a photosensitizer. It can not only be used as a probe for detecting cancer cells but also for the intracellular quantification and diagnosis of other biomolecules, which is an interesting possibility (Fig. 10c). At present, the potential of NCNAs in the field of cancer diagnosis and treatment is only the tip of the iceberg [88]. In terms of cancer treatment strategies, we believe that NCNAs with multiple therapeutic mechanisms will be the future trend and that the use of NCNAs to achieve codrug delivery has broad application prospects.

### Diagnostic logic gates

**Figure 11.**
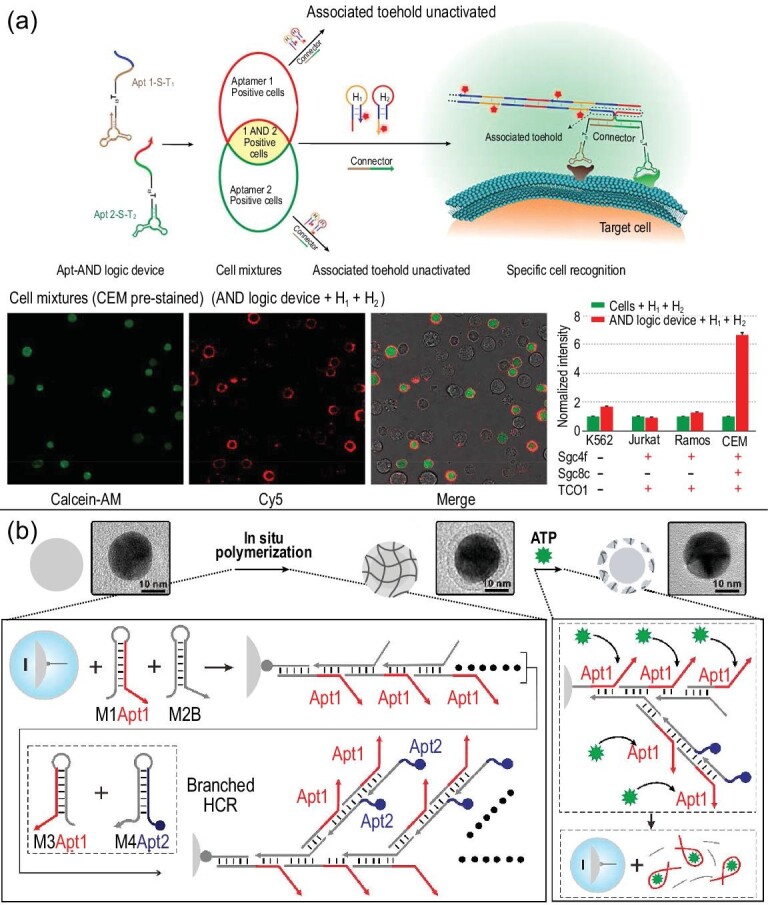
Construction of diagnostic logic gates. (a) Construction of a multiple-aptamer-based DNA logic device on live cell membranes and fluorescence data of different cells. Reprinted with permission from ref. [56]. Copyright 2019, American Chemical Society. (b) Schematic illustration of the preparation of ATP-responsive SNA gel. Reprinted with permission from ref. [35]. Copyright 2019, American Chemical Society.

Through continuous exploration, NCNAs have achieved increased drug delivery efficiency. However, many substances constantly change dynamically during the course of disease and are present in normal cells. This phenomenon hinders the treatment of diseases and calls for logic-based multi-response materials. Nucleic acids are the most promising scaffolds for constructing highly programmable logic devices. They can be rationally designed at the molecular level and are based on gene chains, metal ions, small molecules and receptors. The NCNA body is the input, which is calculated by strand replacement, enzyme cleavage and aptamer recognition, thereby enabling rapid progress in drug delivery, cell imaging, genome editing and information storage [89]. For example, cell targeting mostly uses certain receptors on the cell surface for recognition and targeting. However, the variability and quantity of cell surface materials vary greatly, and a change in one factor may cause changes in cell characteristics. Moreover, normal cells and diseased cells share most cytokines. Therefore, we can logically analyze multiple markers in each subgroup to achieve a more precise identification of individual cell characteristics so that the presence or absence of multiple membrane receptors will enable accurate spectral analysis. A programmable universal platform based on a series of aptamer codes and OR, or non-logic gates, can screen various abnormalities on the surface of the cell, and Boolean operations that support these logical operations can be further programmed to build more complex AND logic systems with higher functionality. Researchers introduced a general method of assembling these modular logic gates to program the identification of multiple coexisting cell surface markers, including multiple markers found on cancer cells, higher-order analysis and diagnostic signal reporting and/or positioning functions [89]. With the rapid development of computer technology, we can design a logic device that can automatically identify and analyze cellular material and mark target cells with markers to accurately identify cells. In addition, some classic cases, such as the multi-aptamer DNA logic device shown in Fig. 11a, can accurately identify cancer cells [56] and analyze for similar cells via the presence or absence of different biomarkers. Xiao's group adopted a hybridization chain reaction to generate SNA gel. Due to a compact DNA shell decorated with aptamers, this SNA gel can prolong blood circulation and improve targeting in the body [35] (Fig. 11b). The application of logic gates enables NCNAs to more accurately identify and treat diseased cells. It is one of the most critical technologies for the future of precision medicine, and it still requires in-depth exploration.

## SUMMARY AND OUTLOOK

This review presents an overview of research into a new type of specific nanocarrier, the NCNA, which has a variety of unique functions that can accelerate many important biomedical applications and address future challenges. We have discussed the latest developments in structural characteristics and capabilities in dynamic enhancements.

Nucleic acids have programmability and sequence specificity, allowing the construction of nanostructures through self-assembly. Therefore, NCNAs can be precisely functionalized, and through sequence changes, they can be integrated with multifunctional molecules such as aptamers, peptides and antibodies to achieve application in the fields of target recognition, biosensing, molecular detection and therapy. In recent years, NCNAs, as a smart material, have led to breakthroughs in diagnostic logic gates that use the programmability of nucleic acids to design NCNAs, use molecules as input, and change dynamically in response to specific molecules or the environment. This NCNA-based computer-aided calculation has high accuracy and is in line with the current demand for high-precision research.

However, the complex structure of NCNAs also means that the effects *in vivo* are difficult to predict. Currently, we do not fully understand the pharmacokinetics of NCNAs, such as circulation, excretion and decomposition, or their physical and chemical properties, such as surface charge and geometry. Moreover, nanocarriers generally rely on endocytosis to enter cells, but cells are always undergoing dynamic changes, and slight changes will change the physical and chemical properties. Therefore, constructing a fully controllable and highly accurate NCNA and reducing non-selective uptake by certain organs and cells has become the focus of recent research.

There is an urgent need for accurate and effective measures in the treatment of major diseases. Although many researchers have made significant contributions [90,91], we are still in the early stages of clinical practice and there is still a long way to go. Researchers should strive to improve the therapeutic effect and detection accuracy of nanomedicine, and maintain biological safety to the greatest extent. In response to these problems, the NCNAs we reviewed exhibit great potential [92–95]. In the long run, our researchers need to achieve a deeper understanding of the characteristics of nucleic acids and further study the construction and application of NCNAs.

All abbrevaviations are summaried in Table 1.

**Table 1.** List of abbreviations.

No.	Full name	Abbreviation
1	Nanocarriers based on nucleic acids	NCNAs
2	Nanoparticles	NPs
3	Spherical nucleic acids	SNAs
4	Rolling circle amplification	RCA
5	Magnetic NPs	MNPs
6	Doxorubicin	DOX
7	Folic acid	FA
8	DNA nano precision-guided missile	D-PGM
9	Liposome spherical nucleic acid	LSNA
10	Immunoadjuvant agents	CpG
11	Dendritic cells	DCs
12	Cell origami clusters	COCs
13	Photodynamic therapy	PDT
14	Photothermal therapy	PTT
15	Indocyanine green	ICG
16	Polo-like kinase I	plk1
17	RNA nanoflowers	RNA NF
18	Near-infrared	NIR
19	Small interfering RNA	siRNA
20	Adenosine tirphosphate	ATP
21	Human antigen R	HuR
22	CCRF-CEM	CEM
23	TdT-mediated dUTP nick-end labeling	TUNEL
24	Hematoxylin-eosin staining	H&E
25	3-Methylglutarylated hyperbranched poly (glycidol)	mGlu-HPG
26	MicroRNAs	miRNA
